# Chronic Psychological Stress in Oncogenesis: Multisystem Crosstalk and Multimodal Interventions

**DOI:** 10.34133/research.0948

**Published:** 2025-10-21

**Authors:** Bin Huang, Honglin An, Han Wu, Yiman Qiu, Yanqing Su, Liming Chen, Vasiliki Epameinondas Georgakopoulou, Jiumao Lin, Wujin Chen, Ruofei Li, Dongliang Yang, Xiaojiaoyang Li, Demetrios A. Spandidos

**Affiliations:** ^1^ The Affiliated People’s Hospital of Fujian University of Traditional Chinese Medicine, Fuzhou, Fujian 350122, China.; ^2^ Academy of Integrative Medicine, Fujian Key Laboratory of Integrative Medicine on Geriatrics, Fujian University of Traditional Chinese Medicine, Fuzhou, Fujian 350122, China.; ^3^ Department of Pathophysiology, Laiko General Hospital, National and Kapodistrian University of Athens, 11633 Athens, Greece.; ^4^ State Key Laboratory of Flexible Electronics (LoFE) and Institute of Advanced Materials (IAM), School of Physical and Mathematical Sciences, Nanjing Tech University (NanjingTech), Nanjing 211816, China.; ^5^ School of Life Sciences, Beijing University of Chinese Medicine, Beijing 100029, China.; ^6^ Laboratory of Clinical Virology, School of Medicine, University of Crete, 71003 Heraklion, Greece.

## Abstract

Malignant tumors constitute a major global public health burden. Chronic psychological stress (CPS) manifests as sustained dysregulation arising from prolonged adaptive responses to chronic endogenous and exogenous stimuli. Clinical evidence indicates that CPS markedly influences cancer progression, with most oncology patients developing detectable stress-related psychological disorders during disease management. This review synthesizes recent advances in understanding CPS-mediated oncogenic mechanisms and evaluates current intervention approaches. Mechanistically, CPS compromises immune surveillance through neuroendocrine-mediated hormonal dysregulation, impairing malignant cell recognition and clearance. Concurrently, CPS hormones promote tumor metabolic adaptation via hypothalamic–pituitary–adrenal axis-driven metabolic reprogramming, enhancing glycolytic flux to support uncontrolled proliferation. CPS further accelerates tumor progression through reactive oxygen species-induced mitochondrial impairment, DNA damage accumulation, and inflammatory cascades. Notably, CPS induces gut microbiota perturbations that reciprocally amplify tumorigenic processes through microbial metabolite disturbances and neuroimmune crosstalk, creating a self-perpetuating pathogenic loop. Therapeutic strategies to address cancer-related CPS that encompass pharmacological agents targeting neuroendocrine pathways, psychosomatic behavioral interventions, social environment adjustments, and evidence-based traditional Chinese medicine formulations demonstrate potential in cancer prevention, treatment, and outcome optimization. However, challenges remain in achieving precise neuromodulation and minimizing intervention side effects, underscoring the need for mechanism-guided therapeutic innovations.

## Introduction

Emotions are complex mental and psychological responses experienced by humans and higher animals in response to changes in both internal and external environments [1]. In traditional Chinese medicine (TCM) theory, this psychological activity primarily centered on emotional states is collectively referred to as the “seven emotions” (joy, anger, anxiety, contemplation, sorrow, fear, and fright) and the “five zhi” (anger, joy, pensiveness, grief, and fear) [2,3]. Excessive emotional states disrupt the normal flow of Qi within the body, leading to a breakdown of Yin–Yang equilibrium and compromised harmony between Qi and blood [4]. Prolonged maintenance of such dysregulation may initiate pathological development or exacerbate existing disease progression. This persistent state of emotional imbalance, which is caused by homeostatic dysregulation stemming from endogenous and exogenous stressors, is termed “chronic psychological stress” (CPS) in modern Western stress theory [5]. CPS elicits persistent negative emotional responses, such as depression, fear, and anxiety [6]. These responses may contribute to substantial severe clinical consequences, particularly in disease pathogenesis. Notably, cancer is recognized as a major pathology associated with CPS [7].

Cancer is a multifactorial disease involving genetic and environmental interactions, consistent with the unified theory of carcinogenesis [8]. This theory attributes carcinogenesis to cumulative genetic and epigenetic alterations in precursor cells, driven by mutational processes and environmental induction of oncogenic pathways through modifiable factors including lifestyle behaviors and CPS [9–11]. While conventional research has predominantly focused on chemical carcinogens and nutritional epidemiology, emerging evidence substantiates CPS as a pivotal modulator of tumor evolution through distinct pathological mechanisms [8,12,13].

The relationship between CPS and carcinogenesis remains controversial. Early epidemiological studies reported no significant correlation between stress exposure and cancer incidence, including meta-analytical evidence dismissing occupational stress as a risk factor for major malignancies [14]. However, contemporary oncology research resolves this paradox: while psychological resilience demonstrates limited capacity to alter cancer mortality, substantial evidence now confirms that persistent CPS states (stress, anxiety, and depression) actively potentiate tumorigenic processes and influence disease prognosis [15]. For example, breast cancer, the second most prevalent malignancy worldwide, frequently induces post-diagnosis psychological distress manifested through depression, anxiety, and perceived stress [16]. The onset of breast cancer itself constitutes a major psychological stressor, triggering clinical anxiety in up to 33.9% and depression in 6.5% of patients at initial diagnosis [17]. Longitudinal studies demonstrate that during or following treatment, 20.2% of breast cancer patients experience anxiety; 45.2% develop depression; approximately 10% exhibit post-traumatic stress disorder; and 14% to 99% report cancer-related fatigue [18,19]. Such CPS patterns markedly impair treatment adherence and elevate recurrence risks and mortality rates, while adversely affecting survival outcomes [20].

Therefore, investigating the biological mechanisms through which CPS regulates cancer progression is critical for advancing our understanding of stress-related disease pathophysiology and developing targeted therapies to improve clinical outcomes. This review synthesizes current mechanistic insights into CPS-mediated cancer development, driving pathophysiological cascades through joint activation of the neuroendocrine system and immune regulatory networks involving immune cells and microbiota, thereby promoting tumor progression and metastasis. Concurrently, we identify therapeutic strategies targeting these specific mechanisms and advocate the systematic integration of emotion-focused interventions into standardized cancer management protocols to optimize treatment efficacy.

## Epidemiological and Preclinical Evidence Validates CPS as a Driver of Tumor Progression

Psychological factors play a critical role in cancer progression and prognosis. Cancer patients frequently experience psychological distress characterized by anxiety, depressive moods, and death-related anxiety, which persists throughout the disease course and evolves into CPS. This psychological state mediates neuroendocrine system imbalance and immune suppression [21], ultimately leading to compromised quality of life and unfavorable clinical outcomes.

### Epidemiological evidence links CPS to oncogenesis and progression

Current research has not yet clearly delineated the differential effects of stress exposure intensity and duration on cancer development in healthy individuals or on accelerated disease progression in cancer patients. Nevertheless, the positive association between CPS and individual cancer incidence as well as cancer progression remains unequivocal. In epidemiological investigations evaluating causal attribution for colorectal cancer, lymphoma, thyroid cancer, endometrial cancer, prostate cancer, and multiple myeloma, 12% of cancer survivors ascribed their malignancy to prolonged stress exposure [22], and approximately 57% of colorectal cancer patients experience depression prior to disease deterioration [11]. Consistent with these observations, studies have demonstrated an association between psychological distress and increased incidence of breast, ovarian, colorectal, lung, gastric, urinary and liver cancer [23,24]. Additionally, clinical evidence indicates a significant association between poor prognosis in cancer patients and severe depression [25]. Multiple cohort studies have established positive correlations between CPS severity (e.g., anxiety and depression) and increased mortality risk in patients diagnosed with colorectal cancer, breast cancer, lung cancer, prostate cancer, liver cancer, bladder carcinoma, and renal cell carcinoma. Depression was associated with a 38% increased cancer mortality risk in mixed cancer types [25,26].

Cancer patients in remission frequently face concerns about potential recurrence, which can lead to CPS and related issues [27], and severe depressive symptoms in breast cancer patients are associated with a 24% increase in the risk of cancer recurrence and 23% higher mortality risk [25,28]. Notably, after patients are diagnosed with late-stage, unresectable cancer, the incidence of cancer-related CPS such as fatigue and emotional disturbances increases significantly [29]. Addressing these patients’ emotional needs is essential for improving their quality of life and may contribute to prolonged survival [30]. Clinical and basic research confirms that psychological counseling or pharmacological interventions are critical for cancer patients, as early antidepressant use not only alleviates depressive symptoms but also increases cancer survival rates [31]. For example, antidepressant combination therapy has demonstrably reduced mortality risk in patients with lung, breast, glioblastoma and colorectal cancers [32,33].

### Direct preclinical evidence links CPS to tumorigenesis

Epidemiological studies investigating the relationship between CPS and cancer development have yielded inconsistent findings, primarily attributed to variations in study designs and statistical methodologies [34,35]. Furthermore, human epidemiological research faces interference from real-world confounding variables and challenges in longitudinal monitoring of stress-related biomarkers, which may obscure causal relationships [36]. To address these limitations, preclinical models using standardized cancer cell lines and controlled stress paradigms (e.g., chronic restraint or social defeat) provide mechanistic clarity, enabling systematic validation of stress-induced oncogenic pathways [37,38].

Notably, CPS promotes hepatocellular carcinoma progression via major histocompatibility complex class I (MHC-I) down-regulation and PD-L1 up-regulation to drive immune escape [39], whereas in breast and pancreatic cancer cases, it induces metastatic outgrowth through glucocorticoid (GC)-activated neutrophil GR signaling that triggers NET-mediated tumor microenvironment (TME) remodeling [21,38]. In non-small cell lung cancer (NSCLC) xenograft models, CPS exposure significantly increased tumor growth, with CPS-exposed nude mice exhibiting a 2.1-fold elevation in tumor volume versus controls [40]. CPS also promotes therapeutic resistance in breast, prostate cancer, and hepatocellular carcinoma through β2-adrenergic receptor (β2-AR)-mediated signaling [23,41]. Investigations in hepatocellular carcinoma models revealed that CPS up-regulates dopamine (DA) synthesis via tyrosine hydroxylase activation and amplifies dopamine receptor type 2 (DRD2) signaling, thereby accelerating tumor progression [41]. In breast cancer models, tumor growth rates and lung metastasis frequency were significantly elevated in stress-induced depressed mice compared to nondepressed counterparts [42,43].

In summary, epidemiological investigations and preclinical studies indicate that sustained negative emotional states may contribute to neoplastic progression. Elucidating the pathophysiological mechanisms linking psychological distress to tumorigenesis has become a pivotal focus in contemporary oncology research. This exploration not only assists cancer patients in enhancing their quality of life and psychological resilience to combat disease progression, but also offers valuable empirical evidence for clinical oncologists to develop targeted therapeutic interventions.

## Multisystem Mechanisms of CPS in Cancer Pathogenesis

**Fig. 1.**
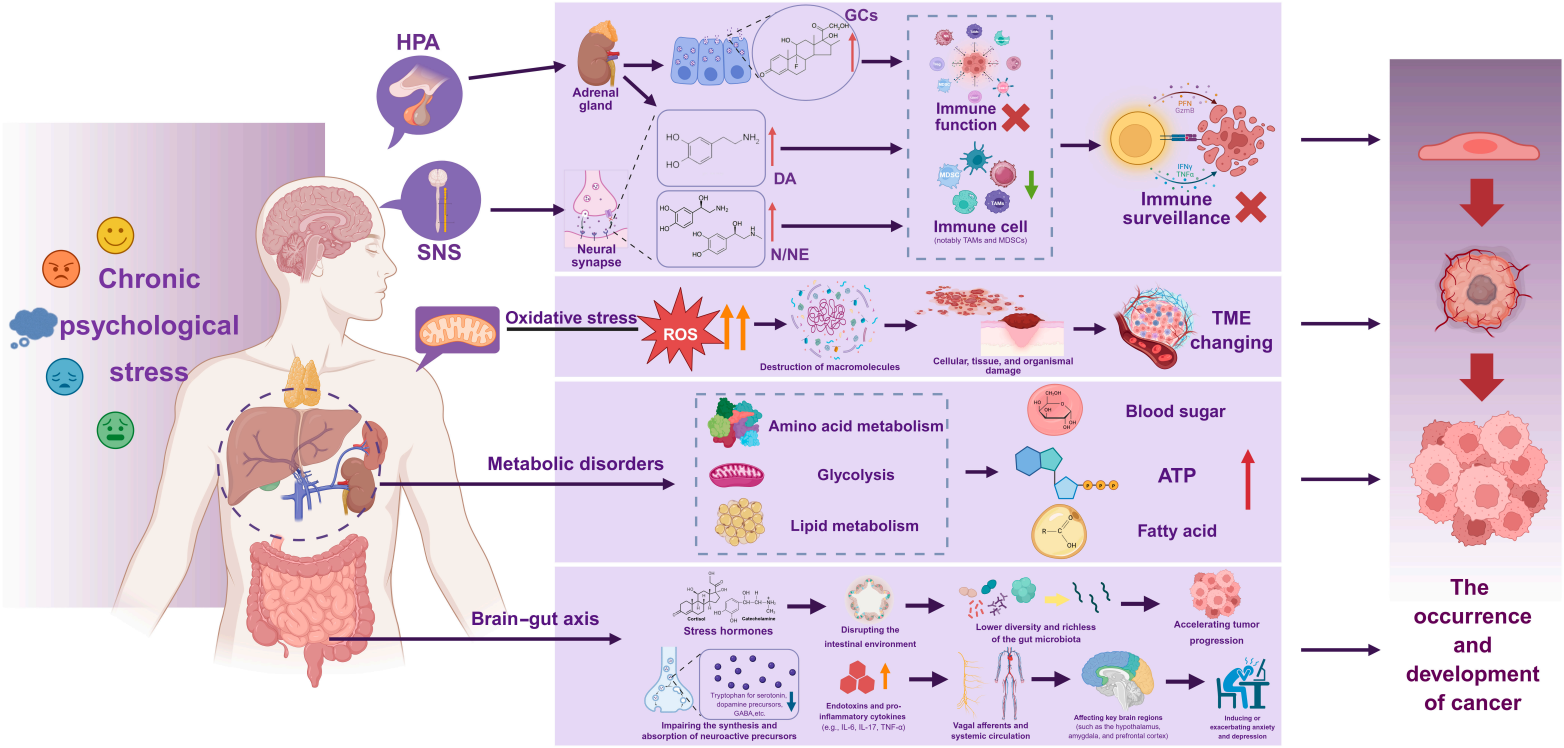
Integrated oncogenic network of chronic psychological stress. CPS overactivates the HPA axis and SNS, dysregulating the release of neuroendocrine mediators (catecholamines/glucocorticoids) that coordinately drive tumor progression through 4 interconnected dimensions: neuroendocrine–immune regulation, oxidative stress imbalance, metabolic reprogramming, and the gut–brain axis, collectively promoting tumor growth, metastasis, and therapy resistance. HPA, hypothalamic–pituitary–adrenal; SNS, sympathetic nervous system; GCs, glucocorticoids; DA, dopamine; N/NE, norepinephrine/ noradrenaline; TME, tumor microenvironment; ATP, adenosine triphosphate.

CPS orchestrates tumorigenesis through multisystem modulation of the TME [44]. CPS-mediated neuroendocrine dysregulation releases catecholamines and GCs that promote tumor proliferation while inducing T cell exhaustion and myeloid-derived suppressor cell (MDSC)-mediated immunosuppression [41,45]. Adrenergically driven oxidative stress disrupts redox homeostasis via reactive oxygen species (ROS) overproduction, stimulating tumor proliferation and metastasis [46]. β2-AR-enforced metabolic reprogramming increases glycolytic flux, driving therapeutic resistance [47]. CPS also induces gut microbiota perturbations that amplify tumorigenesis through microbial metabolite disturbances and neuroimmune crosstalk, forming a self-perpetuating pathogenic loop. This cascade progressively transforms the TME from immune surveillance to pro-metastatic states, establishing a self-sustaining oncogenic ecosystem [23,48]. Collectively, CPS drives a self-perpetuating, therapy-resistant oncogenic ecosystem via multisystem disruption (Fig. 1).

### CPS drives tumorigenesis via neuroendocrine–immune suppression

The immune system preserves physiological homeostasis through vigilant surveillance of cellular alterations and targeted elimination of malignant or dysregulated cells. However, CPS disrupts immune competence via endocrine perturbations. Prolonged sympathetic nervous system (SNS) activation and sustained hyperactivity of the hypothalamic–pituitary–adrenal (HPA) axis under CPS conditions induce aberrant accumulation of stress-related mediators, including DA, GCs, epinephrine/adrenaline (E), and norepinephrine/noradrenaline (NE), within the TME [49–51]. These mediators impair the effector functions of immunocompetent cells such as T lymphocytes, natural killer (NK) cells, dendritic cells (DCs), and macrophages, while concurrently expanding immunosuppressive cellular subsets, notably tumor-associated macrophages (TAMs) and MDSCs [52,53]. This dual mechanism compromises immune surveillance, resulting in diminished capacity to recognize and eradicate transformed cells, thereby facilitating tumorigenesis and malignant progression [21].

CPS triggers aberrant activation of the HPA axis and SNS, leading to dysregulated DA release in both central and peripheral microenvironments [54]. This pathological DA signaling remodels the tumor immune landscape through a multidirectional receptor network involving DRD1 to DRD5. Hyperactivation of some of these receptors suppresses the mTORC1 pathway in CD8^+^ T cells, thereby impairing interferon-γ (IFN-γ) production and granzyme B expression [55], while affecting cyclic adenosine monophosphate (cAMP) phosphorylation in NK cells to diminish tumoricidal activity [56]. In parallel, DA engages DRD1/PI3K signaling to promote T follicular helper (Tfh) cell differentiation [57] and enhance arginase-1 (Arg1)/inducible nitric oxide synthase (iNOS) expression in MDSCs [58]. Concurrently, DRD2-driven MDSCs polarize TAMs to an M2 phenotype, which secrete interleukin-10 (IL-10) and transforming growth factor-β (TGF-β) to establish an immunosuppressive TME [59]. DA is implicated in intratumoral angiogenesis, and low-dose endogenous DA notably suppresses VEGF and inhibits tumor progression via DRD2 activation [60]. Furthermore, DRD5 activation promotes CD103^+^ tissue-resident memory cell differentiation in CD8^+^ T cells and sustains their effector functions, bolstering antitumor immunity and improving clinical outcomes [61].

Compared with early-stage cancer patients and healthy populations, clinical prognosis among advanced cancer patients is exacerbated by anxiety and depression due to subsequent treatment or rapid progression of the disease, accompanied by a sustained release of GCs from HPA secretion, which inhibits the activation and proliferation of immune cells, weakening cytotoxicity and inducing the development of tumor cells [50,62]. GCs can affect the number of CD4^+^ T cells to promote the differentiation of tumor stem cells [63]. The differentiation of CD8^+^ T cells within tumors is influenced by GCs, leading to the exhaustion of CD8^+^ T cells and damaging the body’s immune surveillance function against the tumor [64,65]. Simultaneously, the endogenous signaling of GC affects CD8^+^ T cells, thereby impairing the efficacy of immune checkpoint blockade therapies [64]. Furthermore, stress-related elevation of GCs enhances TAM/C-X-C motif chemokine ligand 1 (CXCL1) signaling, which in turn recruits splenic MDSCs via C-X-C motif chemokine receptor 2 (CXCR2) and creates environmental conditions for breast cancer metastasis [53]. High levels of GCs during depression inhibit the function of tumor-infiltrating NK cells and promote hepatocellular carcinoma progression [66]. Simultaneously, neuroendocrine–immune dysregulation exacerbates hepatic inflammation and fibrosis, while suppressing immune surveillance, thereby promoting the progression from liver fibrosis to hepatocellular carcinoma [67].

Adrenaline receptors critically mediate the interplay between psychological stress and cancer progression through catecholamine-driven mechanisms. CPS persistently activates the SNS, triggering sustained release of epinephrine (E) and norepinephrine (NE), which exert oncogenic effects via adrenergic receptor signaling [68]. β-Adrenergic receptors (β-AR), particularly β2-AR, serve as key mediators: NE binding to β-AR induces CD8^+^ T cell dysfunction and impaired tumor infiltration, contributing to anti-programmed cell death protein 1 (anti-PD-1) resistance in lung adenocarcinoma and gastric cancer cases [69], while β-AR activation broadly suppresses CD8^+^ T cell metabolism in melanoma and colon cancer models [70]. E demonstrates dual functionality, directly enhancing breast cancer stemness via lactate dehydrogenase A (LDHA) regulation in tumor cells while simultaneously suppressing immune responses through β2-AR-dependent mechanisms [71]. These include metabolic reprogramming of MDSCs and inhibition of DC maturation via CD40 signaling pathway disruption [72]. Furthermore, stress-induced E secretion promotes MDSC recruitment through CXCL5-CXCR2-Erk signaling activation and establishes an immunosuppressive microenvironment via COX-2-dependent pathways during myeloma progression [66]. This integrated axis highlights how stress-induced catecholamines coordinate tumorigenic processes through receptor-mediated modulation of both malignant cells and immune components within the TME.

**Fig. 2.**
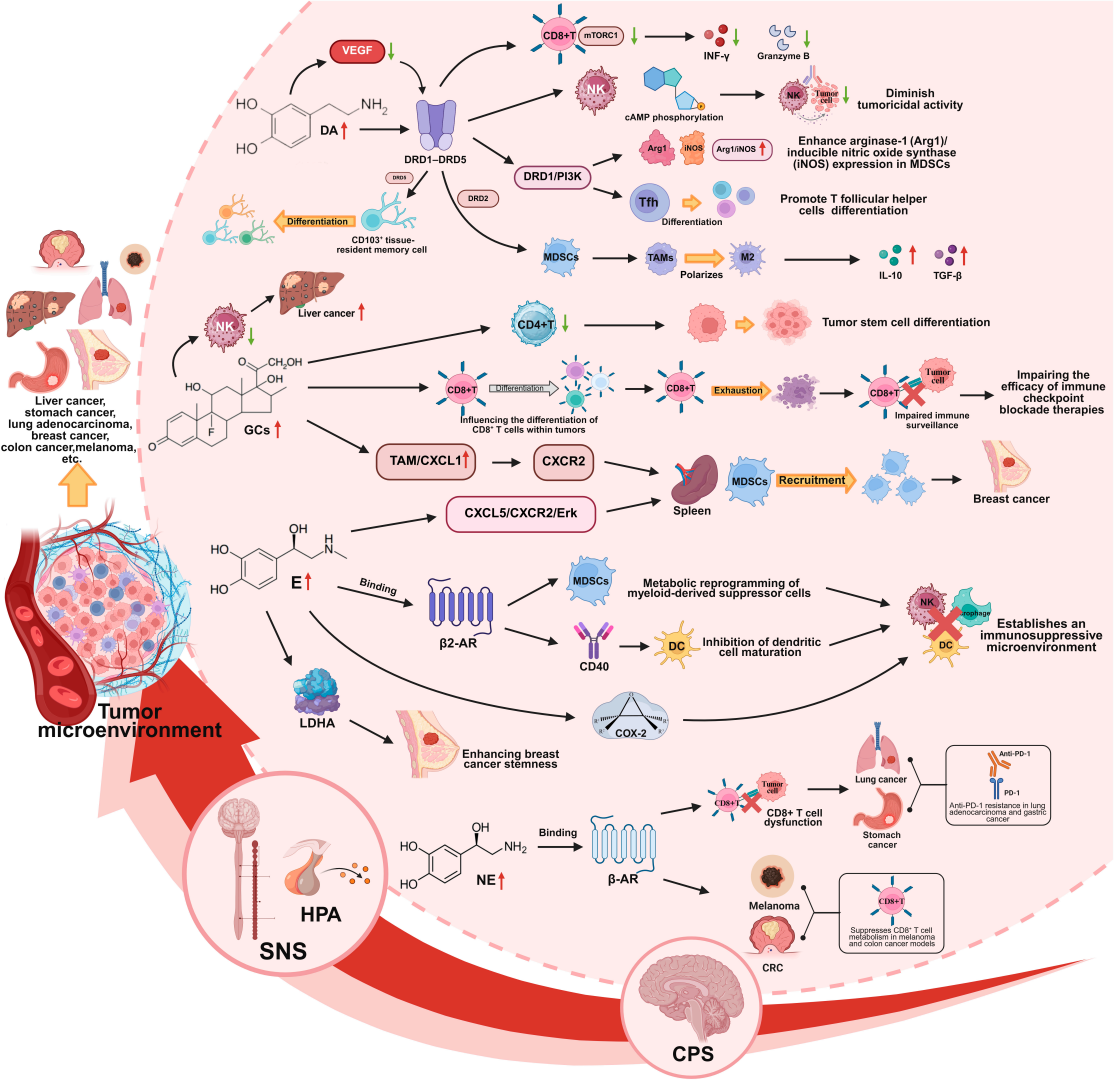
Molecular mechanisms of CPS-induced neuroendocrine–immune suppression promoting tumor progression. CPS overactivates the HPA axis and the SNS, leading to the sustained release of stress mediators (DA, GCs, E, and NE) within the TME. These mediators orchestrate immunosuppression through multiple mechanisms. This integrated neuroendocrine–immuno-oncogenic cascade establishes an immunosuppressive TME that compromises immune surveillance, fosters malignant tumor progression, and contributes to therapy resistance. CPS, chronic psychological; HPA, hypothalamic–pituitary–adrenal; SNS, sympathetic nervous system; DA, dopamine; NK, natural killer cell; IFN-γ, interferon-γ; MDSCs, myeloid-derived suppressor cells; Arg1, arginase-1; iNOS, inducible nitric oxide synthase; Tfh, T follicular helper; TAMs, tumor-associated macrophages; IL-10, interleukin 10; TGF-β, transforming growth factor-β; GCs, glucocorticoids; CXCL, C-X-C motif chemokine ligand; CXCR, C-X-C motif chemokine receptor; Erk, extracellular regulated protein kinases; E, epinephrine; DC, dendritic cell; LDHA, lactate dehydrogenase A; NE, norepinephrine/ noradrenaline; CRC, colorectal cancer.

In summary, CPS promotes tumor development by mediating the release of hormones, the activation of receptors, and the regulation of signaling transduction pathways, which in turn interfere with the activity, proliferation, and cytotoxicity of immune cells, affecting immune surveillance and immune killing functions (Fig. 2). These findings provide clinical evidence for the existence of biomarkers related to CPS, the neuroendocrine system, and tumor progression, such as hormone levels, neurotransmitters, and inflammatory markers. This has substantial implications for the development of drugs that can modulate CPS in cancer patients to synergistically treat tumors and helps to optimize strategies for tumor immunotherapy.

### CPS accelerates cancer growth by altering oxidative stress dynamics

Oxidative stress, resulting from an imbalance between the body’s oxidative and antioxidant systems, leads to the excessive production of ROS [73]. Interestingly, ROS sometimes play a dual role in cancer progression [74]. In the endoplasmic reticulum, peak hepatocellular carcinoma cell viability has been reported at 5 μM H_2_O_2_ (a nonradical ROS), whereas concentrations exceeding 50 μM lead to declined viability. This may be attributed to high levels of ROS inducing oxidative stress, resulting in DNA damage and apoptosis [75]**.** Intracellular ROS act as secondary messengers in signaling cascades, inducing and maintaining the oncogenic phenotype of cancer cells. This can trigger a chain reaction that damages macromolecules such as lipids, proteins, and nucleic acids, ultimately causing cellular, tissue, and organismal damage [76]. Notably, a diminished antioxidant capacity is recognized as a major contributor to various diseases, with tumors being a key example [77].

CPS activates β-adrenergic receptors (such as β2-AR), increases intracellular cAMP levels, promotes the production of ROS, and exacerbates oxidative stress [78]. Long-term stress can lead to mitochondrial dysfunction, which increases the production of ROS and further exacerbates oxidative stress [79]. CPS can also inhibit the activity of antioxidant enzymes, such as superoxide dismutase (SOD), catalase (CAT), and glutathione peroxidase (GPx), thereby weakening the body’s antioxidant defenses and making cells more vulnerable to ROS damage [80]. As signaling transducers, ROS activate oncogenes and inactivate tumor suppressor genes through redox-sensitive pathways (e.g., nuclear factor kappa-B/mitogen-activated protein kinase [NF-κB/MAPK], growth factor receptor binding protein 2/son of sevenless [Grb2/SOS]-Ras-p38, phosphatidylinositol-3 kinase/protein kinase B/mammalian target of rapamycin [PI3K/Akt/mTOR], extracellular signal-regulated kinase [ERK]/MAPK, and Janus kinase/signal transducer and activator of transcription 3 [JAK/STAT3]), triggering malignant behaviors [81–84]. Additionally, ROS trigger chronic inflammatory responses, facilitating the formation of a TME that supports tumor growth and spread [85,86].

Furthermore, excessive ROS caused by CPS can disrupt the redox homeostasis within the endoplasmic reticulum, leading to the accumulation of a large amount of unfolded or misfolded proteins therein, thereby triggering endoplasmic reticulum stress. The subsequent activation of the unfolded protein response, an originally protective cellular mechanism, is exploited by cancer cells to enhance their survival, dissemination, and resistance to therapy [87].

**Fig. 3.**
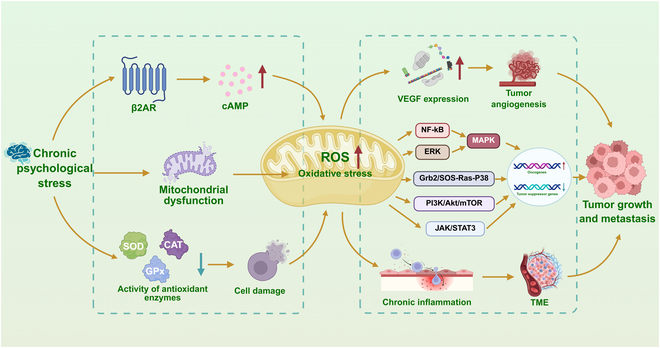
Molecular pathways of CPS-induced oxidative stress imbalance promoting tumor progression. CPS disrupts redox homeostasis through β-AR/cAMP activation, mitochondrial dysfunction, and suppression of antioxidant defenses. The resultant oxidative stress thereby orchestrates pro-tumorigenic effects via oncogenic signaling and TME remodeling. cAMP, cyclic adenosine monophosphate; SOD, superoxide dismutase; CAT, catalase; GPx, glutathione peroxidase; ROS, reactive oxygen species; VEGF, vascular endothelial growth factor; NF-κB, nuclear factor kappa-B; ERK, extracellular regulated protein kinases; MAPK, mitogen-activated protein kinase; Grb2, growth factor receptor binding protein 2; SOS, son of sevenless; Ras, rat sarcoma; PI3K, phosphatidylinositol 3-kinase; Akt/PKB, protein kinase B; mTOR, mammalian target of rapamycin; JAK, Janus kinase; STAT3, signal transducer and activator of transcription 3; TME, tumor microenvironment.

In conclusion, emotional abnormalities induce oxidative stress in the organism, which alters the TME by damaging normal cells and producing cytokines, creating a suitable growth environment for tumors and promoting tumor progression (Fig. 3). Therefore, a more in-depth study of the association between CPS, oxidative stress, and tumor development benefits the development of drugs that regulate CPS from the perspective of resisting oxidative stress in the body, which in turn synergistically prevents and treats tumors.

### CPS disrupts macromolecular metabolism and fuels tumor progression

**Fig. 4.**
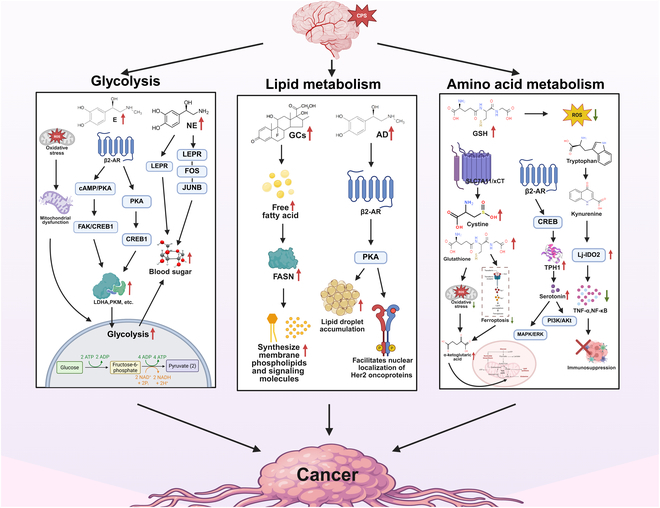
CPS-induced metabolic reprogramming in tumor progression. CPS drives tumor metabolic plasticity through 3 interconnected pathways: glycometabolic reprogramming, lipid remodeling, and amino acid reprogramming, thereby promoting tumor bioenergetics and enhancing therapy resistance. AD, adrenaline; cAMP, cyclic adenosine monophosphate; LDHA, lactate dehydrogenase A; PKM, pyruvate kinase M; NE, norepinephrine; GCs, glucocorticoids; FASN, fatty acid synthase; GSH, glutathione; PKA, protein kinase A; GSH, glutathione; ROS, reactive oxygen species; SLC7A11, solute carrier family 7 member 11; β2-AR, β2 adrenergic receptor; CREB, cAMP-response element binding protein; TPH1, tryptophan hydroxylase 1; MAPK, mitogen-activated protein kinase; ERK, extracellular signal-regulated kinase; PI3K, phosphatidylinositol-3 kinase; Akt, protein kinase B; Lj-IDO2, lotus japonicus indoleamine 2,3-dioxygenase 2; TNF-α, tumor necrosis factor-alpha; NF-κB, nuclear factor kappa-B.

The altered metabolic state represents not only a hallmark of cancer but potentially a fundamental cause. Oncogene-targeted metabolic reprogramming leads to the emergence of aberrant metabolic states [88]. These abnormalities involve critical processes, including glycolysis, amino acid metabolism, and lipid metabolism [89] as shown in Fig. 4.

CPS promotes tumor progression through interconnected neuroendocrine pathways that reprogram tumor metabolism [90]. Stress-induced β2-AR activation drives 3 critical processes: (a) oxidative stress impairs mitochondrial function to force glycolytic dependency, accelerating tumor energy acquisition while depleting normal cell resources [91]; (b) sustained β2-AR signaling confers therapeutic resistance to cancer via cAMP/protein kinase A (PKA)-mediated FAK or cyclic AMP-responsive element-binding protein 1 (CREB1) activation coupled [91,92], which further induces lactate dehydrogenase (LDH) and the rate-limiting enzymes PKM inducing PKM and LDHA expression, promoting lactate efflux that acidifies the TME and enables epidermal growth factor receptor‌ (EGFR) inhibitor resistance [93]; and (c) concurrently, excessive adrenaline activates the β2-AR/PKA/CREB1 pathway to overexpress glycolytic enzymes in colorectal tumors [91].

Parallel endocrine disruptions further fuel tumor metabolism: continuous HPA axis activation elevates GCs, stimulating hepatic glycogenolysis and lipolysis to increase circulating glucose and free fatty acids (FFAs) [94], while norepinephrine activates the leptin receptor (LEPR) and the LEPR–FOS–JUNB pathway to enhance glycolytic flux in cases of liver cancer [95]. These coordinated mechanisms demonstrate how CPS hijacks both receptor signaling and systemic hormone axes to remodel cancer metabolism.

CPS-induced lipid metabolic remodeling directly drives malignant progression. Sustained HPA axis activation releases GCs that elevate circulating FFA levels through adipose tissue lipolysis stimulation [96], supplying abundant lipid precursors for tumor cells [90]. Following uptake, these FFAs fuel fatty acid synthase (FASN)-mediated de novo lipogenesis to synthesize membrane phospholipids and signaling molecules, accelerating the proliferation of malignancies such as colorectal cancer [97]. Concurrent β-oxidation enhancement provides an alternative energy source for the adenosine triphosphate (ATP)-depleted TME [98]. Critically, stress hormones activate the β2-AR/PKA signaling pathway, driving lipid droplet accumulation [99]. This also facilitates the nuclear localization of Her2 oncoproteins [100]. Thus, lipid metabolic dependence not only satisfies tumor bioenergetic demands but also increases metastatic potential via altered membrane fluidity, confirming the fundamental role of metabolic dysregulation in cancer pathogenesis [78,101].

Amino acid metabolic dysregulation critically contributes to stress-associated tumor progression, and CPS coactivates the amino acid transporter SLC7A11/xCT in tumor cells [102] and promotes cystine uptake and glutathione biosynthesis, protecting against both oxidative stress and ferroptosis [103]. Resultant α-ketoglutarate replenishes tricarboxylic acid cycle intermediates, sustaining mitochondrial function during oxidative stress [104]. Simultaneously, glutamine-derived glutathione synthesis increases markedly, scavenging ROS to confer chemotherapy protection [105]. In pancreatic colorectal adenocarcinoma models, β2-AR/CREB-induced TPH1 overexpression promotes serotonin biosynthesis, activating PI3K/Akt and MAPK/ERK cascades [106,107]. Parallel activation of the tryptophan-kynurenine pathway mediates lotus japonicus indoleamine 2,3-dioxygenase 2 (Lj-IDO2) overexpression, markedly reducing tumor necrosis factor-α (TNF-α) and NF-κВ expression and establishing immunosuppressive niches [108]. This redirected amino acid flux supports tumor biosynthetic requirements while stabilizing protumorigenic phenotypes through epigenetic mechanisms such as histone acetylation, providing further evidence for the universal role metabolic rewiring plays in oncogenesis [109–111].

In summary, under CPS, glycolysis, lipid metabolism, and amino acid metabolic pathways form a closely interconnected network through neuroendocrine regulation. For example, catecholamines and cortisol synergistically activate key glycolytic enzymes such as HK2 and LDHA to promote the Warburg effect, or stimulate lipolysis to release FFAs for energy supply, while also enhancing branched-chain amino acid metabolism via the mTORC1 pathway. This metabolic reprogramming not only provides energy and biosynthetic precursors for tumors, but their metabolites also undergo further interconversion through cross-metabolism, collectively shaping a pro-TME that supports tumor progression [112].

### CPS leads to gut microbiota imbalance, promoting tumor development and progression

CPS and gut dysbiosis form a self-perpetuating cycle that fuels tumor development and progression [113]. Serving as the central mediator of the “gut–brain” axis, the gut microbiota engage in bidirectional communication with the brain, influencing tumor progression through 2 primary pathways [114].

CPS (such as depression and anxiety) activates the HPA axis and SNS, releasing stress hormones such as cortisol and catecholamines [115]. These hormones directly disrupt the intestinal environment by impairing motility, mucus secretion, barrier integrity, and local immunity [116]. Consequently, beneficial commensals (e.g., *Lactobacillus murinus*, indole-3-acetate-producing bacteria) decline markedly decline [117], while potential pathogens (e.g., *Clostridium* spp. and *Escherichia coli*) proliferate [118]. The resulting dysbiosis compromises gut barrier function, facilitating the translocation of endotoxins (such as LPS) into the bloodstream. This triggers systemic low-grade inflammation, activates pro-inflammatory signaling pathways (e.g., NF-κB), and polarizes TAMs toward the pro-tumorigenic M2 phenotype, fostering an immunosuppressive microenvironment. This creates conditions for the “inflammation-cancer” transformation in the colon. Concurrently, dysbiosis alters the microbial metabolome, characterized by reduced protective metabolites such as anti-inflammatory and antitumorigenic short-chain fatty acids (SCFAs, e.g., butyrate) and increased levels of pro-carcinogenic metabolites such as secondary bile acids, trimethylamine (TMA), hydrogen sulfide (H_2_S), and tryptophan metabolites [119–121]. These changes collectively promote tumor cell proliferation, inhibit apoptosis, enhance angiogenesis, and increase metastatic potential [122]. Clinically, patients with cancer (including head, neck, breast, and other varieties) or precancerous (e.g., inflammatory bowel disease) lesions and comorbid depression exhibit reduced gut microbial diversity, depletion of specific beneficial taxa, accelerated tumor progression, and poorer prognoses [123,124].

Conversely, gut dysbiosis markedly modulates the central nervous system and host psychological state, thereby influencing tumor progression. Dysbiosis (marked by reduced diversity, loss of beneficial species, and pathogen dominance) impairs the synthesis and absorption of neuroactive precursors (e.g., tryptophan for serotonin, dopamine precursors, and GABA) [114] while elevating circulating levels of pro-inflammatory cytokines (e.g., IL-6, IL-17, and TNF-α) and endotoxins [125]. These alterations affect key brain regions (such as the hypothalamus, amygdala, and prefrontal cortex) via vagal afferents and systemic circulation, inducing or exacerbating anxiety and depression [126]. This further activates the HPA axis, creating a self-reinforcing stress cycle [127]. The ensuing persistent central stress response suppresses antitumor immunity through neuroendocrine pathways (e.g., sustained high cortisol levels impairing NK cell activity and CD8^+^ T cell function) [128]. Additionally, sympathetic nerve-derived norepinephrine directly stimulates tumor cell β-adrenergic receptors, activating downstream pro-oncogenic pathways (e.g., cAMP/PKA) to promote tumor growth, invasion, and metastasis [99]. Experimentally transplanting fecal microbiota from depressed individuals into germ-free mice recapitulates depressive-like behaviors and accelerates tumor growth, while restoring a healthy microbiota improves affective states and suppresses tumors [129].

**Fig. 5.**
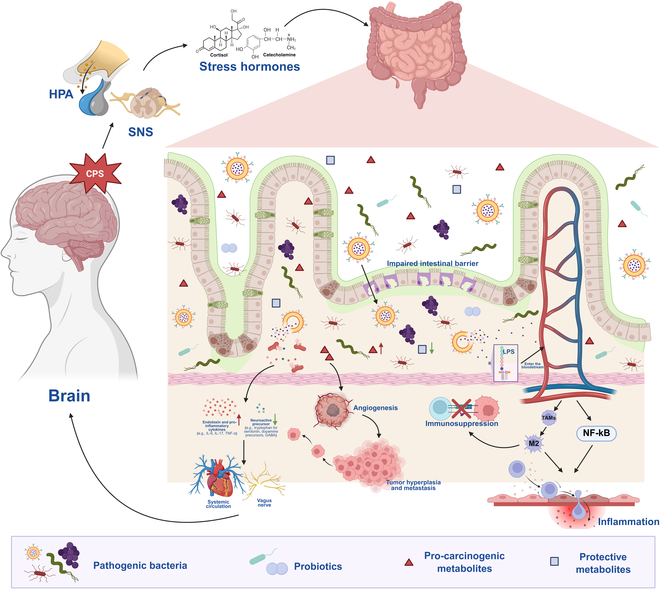
Bidirectional brain–gut–microbiota axis in stress-driven tumorigenesis. CPS activates the HPA axis and SNS, releasing cortisol and catecholamines that compromise intestinal barrier integrity and impair local immunity, thereby inducing dysbiosis. This microbial imbalance facilitates endotoxin (LPS) translocation, triggering systemic inflammation through NF-κB activation and polarizing TAMs toward the M2 phenotype. Concurrently, altered microbial metabolomes reduce anti-tumor SCFAs while elevating carcinogenic metabolites. Furthermore, dysbiosis diminishes neuroactive precursors and increases pro-inflammatory cytokines, which signal via vagal afferents and systemic circulation to exacerbate anxiety/depression and reactivate the HPA/SNS, establishing a self-reinforcing loop. HPA, hypothalamic–pituitary–adrenal; SNS, sympathetic nervous system; NF-κB, nuclear factor kappa-B.

These intertwined “brain–microbiota–tumor” and “microbiota–brain–tumor” pathways form a self-sustaining pro-tumorigenic loop, conceptualized as the stress–microbiome–metabolite–epigenetic–oncology (SMMEO) axis [130–132]. Future research should focus on deciphering the precise mechanisms of bidirectional gut–brain axis regulation (Fig. 5), addressing individual heterogeneity, and developing integrated therapeutic strategies that combine neuromodulation and microbial intervention, which represent promising approaches for synergistic cancer control.

In conclusion, CPS promotes the occurrence and development of cancer by regulating the neuroendocrine system, specifically manifested through the overactivation of the HPA axis and the SNS. This consequently leads to immunosuppression, modulates cancer-related pathways, facilitates the formation of an inflammatory microenvironment, and triggers metabolic reprogramming, ultimately fostering the initiation and progression of cancer. Furthermore, the interaction between the gut–brain axis and the TME should not be overlooked, as they collectively drive tumorigenesis and progression through multiple mechanisms involving neuroendocrine, immune, and metabolic regulation. The CPS-induced pathways across various cancer types are shown in Table 1.

**Table 1.** CPS-driven tumor mechanisms across cancer types

Type of cancer	Key stress-induced pathways	Key mechanisms
Head and neck cancer	1. DA/Teffs pathway	1. DA → DRD2 on CD8^+^ T cells → ↓cAMP → PKA → ↓Rap1 → ↓mTORC1 → ↓IFN-γ and granzyme B → ↓CD8^+^ T cytotoxicity → immune escape [55]
	2. E/β2-AR/CD40	2. NE → β2-AR → ↓IκBα phosphorylation → blocked NF-κB pathway → ↓CD40/MHC-II/CD86 expression → ↓DC antigen presentation → suppressed antitumor immunity [72]
Melanoma	1. DA/MDSCs/iNOS	1. DA → DRD1/DRD5 (D1-like receptors) on MDSCs → ↓ERK/JNK → ↓iNOS → ↓NO → reversal of T cell suppression [58]
	2. GCs/CD8^+^ T	2. GCs → GR on CD8^+^ T cells → ↓TCF-1-dependent effector differentiation, ↑IL-10, ↑PD-1, ↑TIM-3 → dysfunction → tumor immune escape [64,65]
	3. NE/β2-R/CD8^+^ T	3. NE → β2-AR → ↑cAMP → ↑PKA → ↓IFN-γ, ↓GzmB, ↓metabolic energy → ↓CD8^+^ T cytotoxicity [70]
Glioblastoma	DA/TAMs	DA → DRD2 → TAM polarization to M2 → ↑IL-10, ↑TGF-β [59]
Lung cancer	1. DA/VEGF	1. DA → D2R → ↑SHP-2 phosphatase → VEGFR2 dephosphorylation → ↓VEGF/VEGFR2/ERK/AKT signaling → ↓endothelial cell proliferation/migration, ↑apoptosis → ↓angiogenesis/vascular permeability → tumor vascular normalization → chemosensitization/TME remodeling [60]
	2. ROS/JAK/STAT3	2. ROS → STAT3 phosphorylation → nuclear translocation → mitochondrial ROS positive feedback → tumor progression [81]
	3. ROS/PI3K/Akt	3. ROS → c-Met phosphorylation → PI3K → Akt phosphorylation → ↑Bcl-2, ↓BAD → autophagy resistance [82]
Colorectal cancer	1. DA/CD103^+^ TRM	1. DA → DRD5 → cAMP/PKA pathway → ↑RUNX3/HOBIT/NR4A1 → CD8^+^ T differentiation into CD103^+^ TRM cells → ↑IFN-γ/GZMB/TNF-α secretion → ↑tumor cell killing → enhanced immune surveillance [61]
	2. NE/β2-AR/CD8^+^ T	2. NE → β2-AR → ↑cAMP → ↑PKA → ↓IFN-γ, ↓GzmB, ↓metabolic energy → ↓CD8^+^ T cytotoxicity [70]
	3. GCs/FFAs	3. GCs → ↑lipolysis, ↑DNL, ↓β-oxidation → ↑FFAs → ↑membrane phospholipid synthesis, ↑TNF-α/IL-6 signaling → ↑tumor growth [96,97]
	4. β2-AR/CREB/TPH1	4. β2-AR activation → Gαs → ↑cAMP → PKA → CREB phosphorylation → ↑TPH1 → ↑serotonin → 5-HT receptor activation → PI3K/Akt, MAPK/ERK activation → cancer cell proliferation [106,107]
	5. Gut–brain axis	5. Stress hormones → gut microbiota dysbiosis → intestinal barrier disruption → endotoxin translocation → TLR4/NF-κB pathway activation → proinflammatory cytokine release → TAM polarization to pro-tumor M2 phenotype → immunosuppressive TME [122] Stress hormones → gut dysbiosis → ↓protective metabolites (e.g., SCFAs), ↑oncogenic metabolites → HDAC/GPCR inhibition, Wnt/β-catenin activation → ↑tumor proliferation/angiogenesis/metastasis [122]
	6. ROS/VEGF	6. ↑ROS → HIF-1α stabilization → ↑VEGF → ↑proliferation and migration of vascular endothelial cells → tumor angiogenesis [220]
Hepatocellular carcinoma	1. GCs/GR/NK	1. GCs → GR on NK cells → ↓NK cytotoxicity → ↑tumor immune escape [66]
	2. E/COX2/CXCL5/CXCR2/ERK	2. E → β2-AR → ↑COX-2 → ↑PGE2 → ↑CXCL5 → CXCR2 → ERK/MAPK pathway activation → ↑tumor proliferation and migration [53]
	3. NE/LEPR/fos/junb	3. ↑NE → LEPR phosphorylation → FOS → JUNB → ↑AP-1 complex → ↑tumor cell proliferation [95]
Endometrial cancer	β2-AR/cAMP/ROS	β2-AR → AC → ↑cAMP → PKA → ↓SOD, ↑NOX → ↑ROS → mitochondrial dysfunction → ↑ceramide → ROS positive feedback loop [78]
Breast cancer	1. GCs/TAM/CXCL1	1. GCs → GR on TAMs → ↑CXCL1 → CXCR2 → PMN → splenic MDSC recruitment → ↓CD8^+^ T function → ↑cancer metastasis [53]
	2. E/β2-AR/LDHA	2. E → β2-AR → ↑LDHA → ↑lactate → ↓pH → USP28 conformational change → MYC deubiquitination → ↑SLUG transcription → ↑NANOG/OCT4 → ↑breast cancer stemness [71]
	3. Catecholamines/ADAM10/Her2/COX-2	3. Catecholamines → ↑miR-199a-5p and SIRT1 expression → ↑ADAM10 expression → Her2ECD cleavage → γ-secretase-mediated Her2ICD generation → Her2ICD nuclear translocation → ↑COX-2 → ↑tumor metastasis [100]
	4. SLC7A11/xCT/Glutathione	4. SLC7A11 → ↑cystine uptake → ↑glutathione → protection against oxidative stress and ferroptosis [102]
Lymphoma	DA/NK/cAMP	DA → DRD2/DRD3/DRD4 (D2-like receptors) on NK cells → ↑Gi protein → ↓adenylate cyclase → ↓cAMP → ↓PKA → ↓p-CREB → ↓cytotoxicity [56]

## Interventions for CPS in Cancer Patients and Clinical Efficacy

**Fig. 6.**
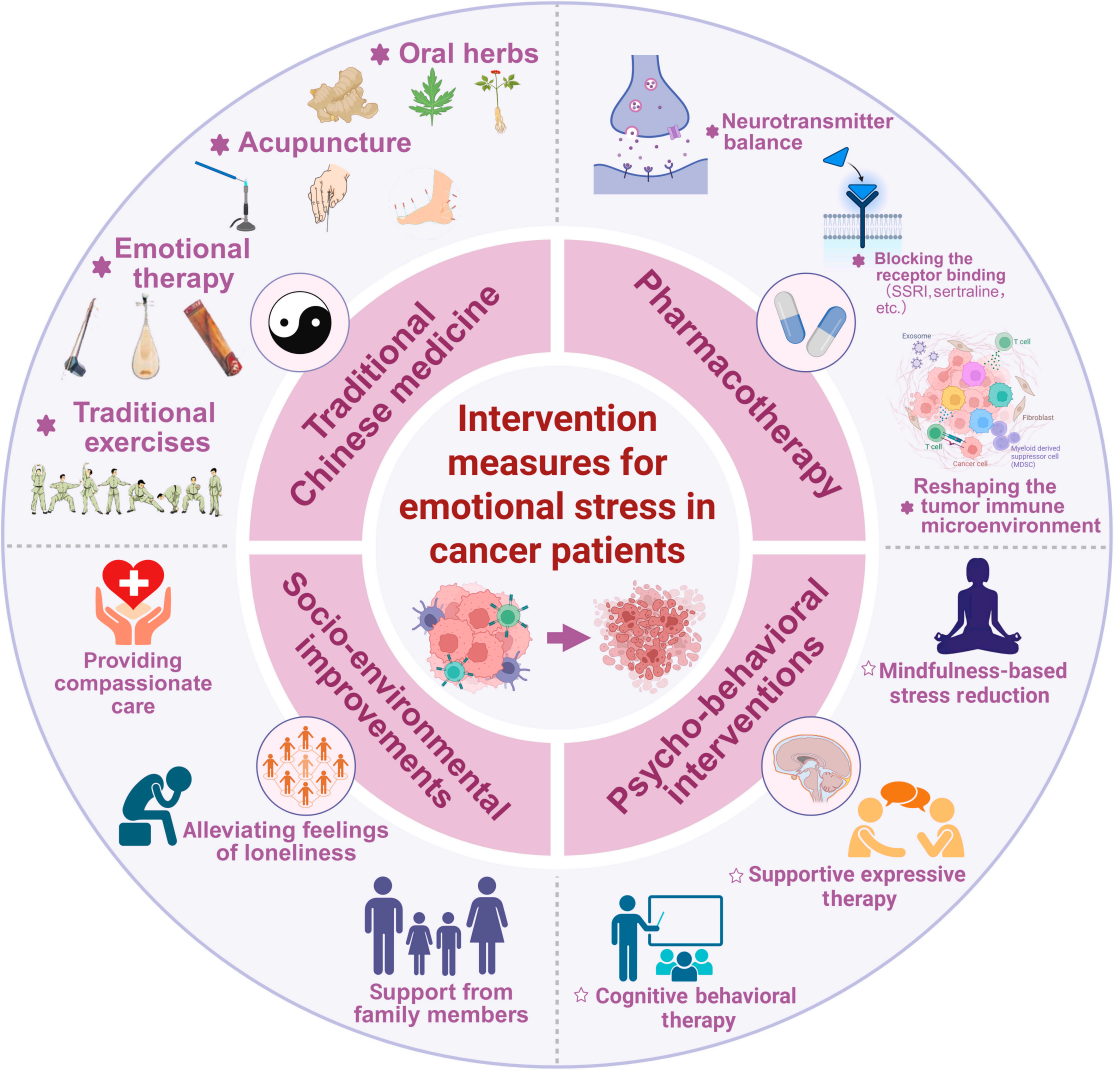
Integrative therapeutic strategies against CPS-driven tumor progression. CPS in cancer patients is therapeutically addressed through 4 integrated dimensions—pharmacotherapy, psycho-behavioral interventions, socio-environmental optimization, and TCM—which collectively normalize neurotransmitter imbalance, suppress inflammatory cascades, reverse TME acidosis, and enhance immune surveillance, ultimately improving survival rates, enhancing treatment efficacy, and elevating patients’ quality of life.

Cancer patients frequently face psychological distress, adversely affecting their treatment adherence and outcomes. Evidence-based interventions address these symptoms and may modulate disease progression. Key approaches include the following: pharmacotherapy (PT), psycho-behavioral interventions (PBIs), socio-environmental improvements (SEI), and TCM. These strategies deliver critical adjunctive support for optimized cancer care (Fig. 6).

### Pharmaco therapy

Neuromodulatory drugs play a dual role in the comprehensive management of cancer patients. They effectively alleviate symptoms of anxiety, depression, and other CPS-related conditions by regulating neurotransmitter balance (such as serotonin and norepinephrine) within the central nervous system, thereby improving patients’ quality of life [31,133]. More importantly, by precisely targeting the critical “neuro-tumor interaction” axis in tumor development, they block the binding of stress hormones to their receptors on tumor cells or within the TME, modulate abnormal neurotransmitter signaling pathways in tumor cells, and reshape the tumor–immune microenvironment disrupted by CPS [134]. This mechanism inhibits tumor cell growth and metastasis, reverses therapeutic resistance (e.g., to chemotherapy, targeted therapies, and immunotherapy), and ultimately enhances treatment efficacy [135].

Antidepressant drugs, while improving the psychological state of cancer patients, have increasingly demonstrated direct antitumor activity [31,40]. Their clinical application has expanded from basic mechanism exploration to multicancer treatment practices. Selective serotonin reuptake inhibitors (SSRIs), as a core category of these drugs, exert antitumor effects through multiple pathways [136]. In inducing tumor cell apoptosis and cycle arrest, fluoxetine activates the ATF4-AKT-mTOR signaling axis, triggering endoplasmic reticulum stress and protective autophagy, which blocks NSCLC at the G0/G1 phase without damaging normal lung epithelial cells [32]. In regulating autophagy and metabolism, SSRIs (e.g., paroxetine) inhibit the autophagic flux, inducing the mitochondria-dependent apoptosis in lung cancer [137]. Regarding reversing multidrug resistance, sertraline can counteract P-glycoprotein function, increasing the accumulation concentration of chemotherapeutic drugs such as oxaliplatin and paclitaxel in solid tumors [138]. Additionally, sertraline can enhance the response rate of glioblastoma to immune checkpoint inhibitors by blocking NADH and ATP generation and restricting nucleotide synthesis [139].

Tricyclic antidepressants also exhibit unique mechanisms: imipramine overcomes therapy resistance by dually blocking cell cycle progression and DNA repair pathways in breast cancer, enhancing PARP inhibitor efficacy in preclinical models [140]; amitriptyline regulates tryptophan metabolism by antagonizing TLR4 and serotonin transporter (SERT), enhancing the sensitivity of epithelial ovarian cancer to immune checkpoint blockade therapy [141]. Clinical studies have confirmed the synergistic effects of these drugs with conventional therapies [142]. SSRIs combined with PD-1 blockade enhance T cell antitumor immunity and suppress tumor growth in multiple cancer types by inhibiting SERT to restore intratumoral T cell-autocrine serotonin signaling [49]. Sertraline reverses CD8^+^ T cell dysfunction in CPS via normalized IFN-γ/GzmB expression and reduced PD-1, boosting anti-PD-1 efficacy [143]. Notably, SSRIs alone or in combination regimens have antitumor effects on lung cancer, colorectal cancer, breast cancer, etc., achieving the dual benefits of psychological intervention and physiological treatment, especially in tumor patients with comorbid depression [32]. The systematic evidence chain from molecular mechanisms to clinical efficacy provides theoretical and practical support for integrating antidepressants into comprehensive cancer treatment strategies.

Beta-adrenergic receptor blockers, such as ADRB1, have progressed from preclinical research to clinical validation in oncology [144,145]. Studies have elucidated their multifaceted mechanisms: by inhibiting the β2-AR signaling pathway, these agents not only directly suppress the proliferation of triple-negative breast cancer and oral squamous cell carcinoma cells but also markedly reverse multidrug resistance in the case of various tumors [146,147]. For instance, propranolol overcomes sorafenib resistance in hepatocellular carcinoma [148]. In soft-tissue sarcomas (leiomyosarcoma, liposarcoma, angiosarcoma, and solitary fibrous tumor), propranolol synergistically inhibits multidrug resistance and survival pathways to overcome β-adrenergic signaling-linked chemotherapy resistance, demonstrating enhanced tumor control with doxorubicin or docetaxel in preclinical evidence [149]. Additionally, ICI-118,551 inhibits transactivation of the EGFR-Akt/ERK1/2 pathway in colorectal cancer to impede tumor progression [150]. The novel β3-AR antagonist SR59230A has also been validated to suppress hypoxic myeloid leukemic cell survival and doxorubicin resistance [151]. Systematic research evidence highlights that beta-blockers disrupt adrenergic signaling—a critical node in tumor progression and drug resistance—thus establishing a scientific foundation for their integration into comprehensive cancer treatment strategies.

Simultaneously, several emerging neuromodulatory strategies have demonstrated multitarget regulatory advantages in oncology. The α2-adrenergic receptor agonist clonidine, by directly activating host macrophages and enhancing T lymphocyte function, effectively reshapes the immunosuppressive TME [152]. When combined with immune checkpoint blockade, this optimized clonidine treatment markedly boosts antitumor immunity and overcomes resistance in melanoma models [153]. The GC receptor antagonist mifepristone exhibits dual functions in intracranial tumor models: by targeting the GC receptor–CCR8 axis, it both abrogates GC-induced T cell sequestration in bone marrow and enhances antitumor immunity by disrupting CCR8-mediated T cell recruitment [154]. High-dose vitamin C, functioning as a potent enhancer of tumor suppressor TET2 activity, activates TET2 demethylase to up-regulate chemokine expression and hydroxymethylate immune-related genes (e.g., TH1-type chemokines), simultaneously inhibiting PD-L1 up-regulation to enhance tumor-infiltrating lymphocyte infiltration and CD8^+^ T cell function [155].

The interactions between these neuromodulatory agents and standard oncology treatment regimens are crucial for achieving synergistic effects. For instance, β-blockers have been demonstrated to enhance the efficacy of numerous chemotherapeutic agents [156]. In radiotherapy, blocking β-adrenergic signaling can mitigate side effects and exert radiosensitizing effects [157]. Regarding synergy with immunotherapy, aforementioned SSRIs and β-blockers can improve T cell function and remodel the immune microenvironment, thereby overcoming resistance to immune checkpoint inhibitors and improving response rates [31,135].

We have integrated the neuroendocrine and immunometabolic mechanisms of CPS to propose a clinical stratification framework from the following 3 perspectives: (a) Stratification based on neuroendocrine biomarkers (e.g., cortisol, catecholamines, and IL-6): patients with elevated levels may preferentially receive β-blockers (e.g., propranolol) to alleviate immunosuppression, or SSRIs (e.g., escitalopram) to remodel the immune microenvironment; (b) Guidance by tumor molecular subtype: β-blockers may be suitable for triple-negative breast cancer patients; PD-L1 high-expressing tumors may benefit from SSRIs combined with immunotherapy; and chemotherapy-resistant patients may be recommended a regimen of propranolol plus sorafenib; (c) Assessment of comorbidities and concomitant medications: for instance, combining SSRIs in patients with anxiety or depression tendencies and adding β-blockers in those with elevated cardiovascular risk, so as to achieve precision intervention.

### Psycho-behavioral interventions

In oncology clinical practice, psychosomatic behavior interventions regulate the vicious cycle of “stress–neuroendocrine dysfunction–tumor progression” through multitarget modulation, dynamically adjusting stress responses and enhancing vagal tone. These interventions synergistically optimize psychophysiological indices, markedly ameliorate emotional stress, and improve therapeutic outcomes in cancer patients. Psychotherapy, as the core strategy, includes cognitive behavioral therapy (CBT) [158], mindfulness-based stress reduction (MBSR) [159], and supportive expressive therapy [160]. By restructuring negative cognition, meditation training, and emotional catharsis, these therapies effectively suppress HPA axis activity, reducing cortisol levels [161,162]. For instance, in randomized controlled trials (RCTs) of breast cancer patients, the MBSR group demonstrated decreased inflammatory markers (CRP/IL-6), alleviated anxiety and depression, improved sleep quality, reduced opioid dosage in advanced stages, and enhanced chemotherapy completion rates along with better quality-of-life scores [163,164].

Innovative technologies such as digitally delivered mindfulness apps (e.g., CanRelax 2) achieve clinically meaningful distress reduction in cancer patients, while virtual reality-integrated neurofeedback demonstrates marked anxiety improvements across multiple treatment sessions [165]. Exercise interventions, benchmarked at 150 min of weekly aerobic exercise combined with resistance training, inhibit the β-adrenergic receptor/cAMP pathway, reduce LDH secretion to reverse TME acidification, decrease adrenaline levels, and increase CD8^+^ T cell infiltration, thereby reducing 5-year postoperative recurrence rates of breast cancer [166,167]. Mind–body practices (e.g., Yoga and Qigong) further strengthen vagal modulation and heart-rate variability, decreasing salivary cortisol, markedly relieving cancer-related fatigue, and prolonging progression-free survival [168–170]. These interventions collectively target the neuro-immune-metabolic axis: from suppressing pro-inflammatory cytokines (reduced IL-6/TNF-α) to activating antitumor immunity, and from reversing microenvironmental acidosis to improving treatment tolerability, enhancing both survival benefits and quality of life.

Notably, the selection of psychological interventions must account for key clinical practice challenges, such as developing personalized psychological interventions for patients, designing interventions that achieve synergistic effects with pharmacological treatments, and addressing patient adherence issues [171].

### Socio-environment improvement

Patients’ social environment support is often overlooked in cancer management, yet it is crucial for treatment outcomes and survival. Many cancer patients frequently face issues of social isolation and reduced social support, which not only bring substantial psychological distress (such as depression and anxiety) but also have a direct impact on physiological well-being. For example, colorectal cancer patients often lack social support and are isolated [172]; decreased social support among lung cancer patients is associated with an increased risk of death [173]; and breast cancer patients often experience poor sleep and psychological depression [174], while social isolation itself increases their incidence and mortality rates [175]. This CPS caused by social isolation can exacerbate complications, promote tumor activation, and increase mortality [176,177]. The biological mechanisms primarily involve the continuous activation of the HPA axis [176], inflammatory storms, and stress-related physiological changes (such as elevated enkephalins and endorphins levels) due to social isolation [178]. Therefore, effectively reducing social isolation, alleviating feelings of loneliness, and providing compassionate care, especially critical support from family members, partners, and spouses, are of special significance for enhancing patients’ ability to cope with stress, improving quality of life related to physical and mental health, and even improving tumor treatment outcomes.

In oncology clinical treatment, SEI achieves multidimensional synergy by reconstructing the “individual–family–society” support chain [179]. Strengthening core family support serves as the fundamental approach [180]. For instance, implementing the Relationship Intimacy Model for spouses of patients with cancer can reduce catecholamine release related to family conflicts and lower salivary cortisol levels, thereby decreasing patients’ pain scores and opioid medication dosage, and more markedly reducing the 5-year recurrence risk [181,182] Upgrading community support networks is a critical measure [183]. Through structured patient support groups and artificial intelligence (AI)-driven digital connections (which intelligently match “cancer-fighting partners” across different cancer types), improvements in social identity and real-time emotion monitoring lead to greater reductions in depression scores compared to monotherapy, shorter relief time for social anxiety, increased survival rates, and decreased treatment interruption rates [184,185]. Integrating social policies provides safeguarding measures. Flexible working systems post-cancer surgery maintain patients’ social role recognition, resulting in higher return-to-work rates, reduced fatigue [186], and decreased 5-year recurrence risks [187]. These multilevel social environment interventions ultimately produce 3 clinical benefits: first, improved treatment tolerability (reduced pain [188], decreased opioid usage [189], and lower treatment interruption rates [190]); second, optimized quality of life (improved sleep, elevated social function scores, increased family satisfaction, and higher return-to-work rates) [186,191,192]; and third, markedly enhanced prognosis [193]. In metastatic cancer, this includes extended median survival, increased 3-year survival rates, and reduced recurrence risks, establishing these interventions as evidence-based pillars of oncological social prescription [194].

### Traditional Chinese medicine

In tumor clinical treatment, TCM serves as an important adjuvant, enhancing therapeutic efficacy through its core concept of “simultaneous treatment of body and spirit” [195,196], to alleviate treatment-related toxicities from chemo/radiotherapy. Agents such as Babao Dan exemplify this approach, achieving “toxicity reduction and efficacy enhancement” while improving patients’ quality of life [197]. This involves multidimensional interventions for emotional stress (e.g., anxiety, depression, and fear). TCM posits that “emotional injuries from the Seven Affects” are key factors in tumorigenesis and progression [198]. Moreover, tumor diagnosis and treatment themselves exacerbate emotional imbalances, creating a vicious cycle. Based on holism and syndrome differentiation principles, TCM employs comprehensive methods—including oral herbs, acupuncture, traditional exercises, and emotional therapy—to harmonize Qi and blood, balance Yin and Yang, soothe the liver, relieve depression, and calm the mind [199,200]. This approach breaks the negative feedback loop between emotions and tumors, improving patient disease resistance and treatment tolerance [196].

Modern pharmacological studies have shown that TCM ameliorates CPS and related disorders through multitarget mechanisms and holistic regulation. Its mechanisms of action involve modulating key signaling pathways such as BDNF-TrkB, PI3K/Akt/mTOR, and MAPK, alleviating hippocampal neuronal damage [201], and synergizing with brain-derived neurotrophic factor to enhance cellular autophagy, thereby restoring neural plasticity [202]. Additionally, TCM exerts antidepressant effects by regulating the gut microbiota structure and suppressing inflammatory responses [203]. Notably, the regulatory influence of TCM on the neuro–immune–gut network may extend to the prevention and treatment of cancer induced by CPS. It can inhibit tumor initiation and progression by improving gut–brain axis function and reducing the release of inflammatory factors [204].

Compared with Western medicine, TCM emphasizes the holistic concept and treatment based on syndrome differentiation, linking emotional disturbances (e.g., “anger impairs the liver, excessive joy harms the heart”) to zang-fu organ functions. Through personalized regulation, TCM aims to restore mind–body balance at its root rather than merely controlling symptoms. Furthermore, TCM employs natural herbs, acupuncture, and other therapies that avoid common drawbacks of Western drugs—such as gastrointestinal reactions from antidepressants and dependency risks of sedatives—due to their nonaddictive nature and minimal side effects [205].

Multiple guidelines have highlighted the core value of the theory of emotional pathogenesis in TCM for the prevention and treatment of related diseases. With pattern pathogenesis as the central focus, treatment principles, formulations, and administration techniques are flexibly applied accordingly [206,207]. CPS leading to pathological states such as qi stagnation, blood stasis, and phlegm congealment is considered an important foundation for the development of tumors [208]. Clinically, the common treatment principles are to soothe the liver to relieve depression and to harmonize qi and blood [209], thereby blocking the transmission chain of “emotion–qi movement–phlegm and stasis–cancerous toxin” [210]. For example, for patients with liver depression and spleen deficiency, modified Xiaoyao San can markedly improve stress-induced dysfunction of the HPA axis, reduce cortisol levels [211], while also regulating immunoglobulin expression and restoring immune surveillance function, thereby inhibiting the formation of the TME [212]. For those with qi and blood deficiency combined with stasis and stagnation, the use of Qigui Tongluo Oral Liquid combined with moxibustion at Shenque can improve somatization symptoms such as fatigue and anxiety, further reducing oxidative stress damage [213] and slowing tumor cell proliferation [214].

Nonpharmaceutical TCM therapies are also crucial. Acupuncture with electroacupuncture stimulates the dorsal nucleus of the vagus nerve, enhancing NK cell activity [215]. Ear acupoint pressing rapidly reduces anxiety and improves the treatment effect and quality of life of gastric cancer patients [216]. Ba Duan Jin movements (e.g., “Shaking the Head and Swinging the Tail to Clear Heart Fire”) and Five-Element music therapy (e.g., Jiao tune) are better for physical function, quality of life, symptoms, pain, and mental health indicators [217]. Collectively, these improve cancer patients’ pain thresholds and immune function. Thus, integration of TCM and Western medicine enhances cancer pain tolerance and immune competence, thereby improving efficacy in clinical cancer prevention and treatment [218,219]. The emotion regulation strategy central to TCM is indispensable for optimizing patient outcomes.

## Conclusion

**Fig. 7.**
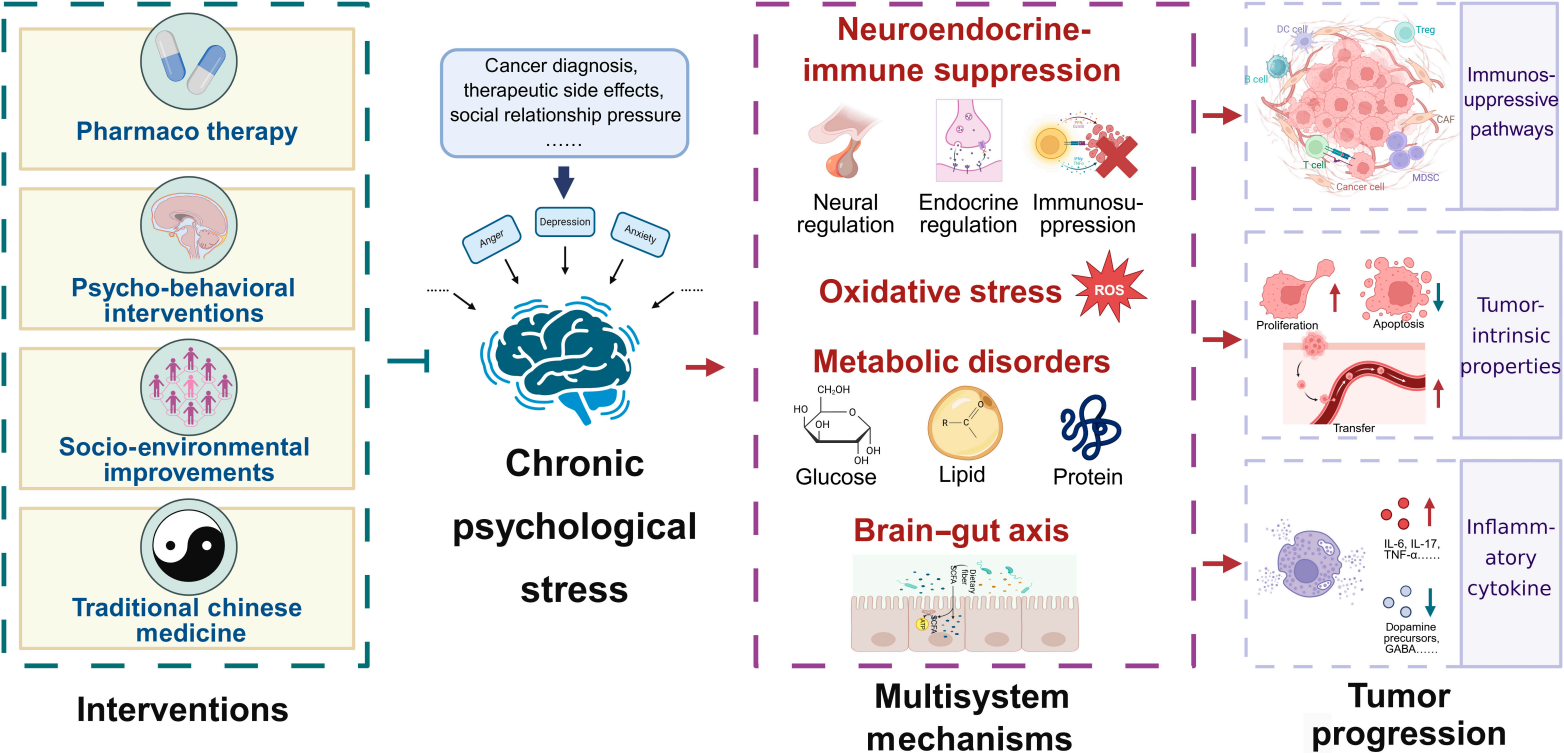
CPS in oncogenesis: Multisystem crosstalk and multimodal interventions. CPS collectively promotes tumor initiation and progression through neuroendocrine–immune dysregulation, oxidative stress imbalance, metabolic reprogramming, and the gut–brain axis. To address these complex mechanisms, multimodal intervention strategies are adopted, including pharmacological therapy, psychological and behavioral interventions, social and environmental improvements, and traditional Chinese medicine. IL-6, interleukin-6; IL-7, interleukin-7; TNF-α, tumor necrosis factor-α.

The multifaceted role of CPS in tumorigenesis underscores the necessity for multimodal therapeutic strategies targeting its complex mechanisms (Fig. 7). CPS is a critical modulator in tumor initiation and progression, engaging in a complex bidirectional pathological interplay. This relationship is mediated through interconnected neuroendocrine–immune–oxidative stress–metabolic–gut microbiota networks, markedly influencing tumor development, metastasis, and therapeutic response. Mechanistically, CPS activates the HPA axis and SNS, driving sustained release of cortisol and catecholamines that suppress CD8^+^ T/NK cell surveillance, while functional changes in MDSCs and TAMs create an immunosuppressive microenvironment. Concurrently, CPS induces oxidative stress via β2-AR/cAMP pathways, increasing ROS accumulation and activating pro-oncogenic signaling. It further reprograms tumor metabolism through β2-AR/PKA/CREB axes to enhance glycolysis and biomass synthesis, while disrupting gut microbiota to reduce antitumorigenic SCFAs and enable endotoxin translocation—forming a self-reinforcing oncogenic cycle via the gut–brain axis.

Multidimensional interventions demonstrate synergistic antitumor potential. PT with SSRIs and β-blockers directly modulates neurotransmitter imbalances and blocks stress hormone signaling to reverse immunosuppression and overcome treatment resistance, though systemic side effects and response variability remain limitations. PBIs, including CBT and mindfulness, effectively inhibit HPA hyperactivity, reduce inflammatory cytokines, and enhance immunity, but face challenges in resource intensiveness and standardization. Through family/community support, SEI mitigates isolation-related physiological harm and improves treatment tolerability, albeit with context-dependent effectiveness. TCM employs herbal formulas, acupuncture, and mind–body exercises under its “adjusting mental state to treat cancer” principle, modulating neurotransmitters and TMEs with good tolerability, yet broader adoption is hindered by limited mechanistic validation and RCT evidence.

Critical challenges persist despite progress. Individual heterogeneity in stress responses and tumor susceptibility, particularly within the SMMEO axis, demands further mechanistic elucidation. Key priorities include developing multiomics-based biomarker systems (hormone–metabolite–microbiome profiles) for precision interventions, conducting high-quality longitudinal trials to validate long-term survival benefits, and optimizing resource-efficient delivery models for psychological support and SEI—especially in resource-limited settings. The future lies in personalized, multimodal integration of PT, PBI, SEI, and TCM tailored to individual stress phenotypes, tumor biology, and social contexts. Leveraging AI for risk stratification/digital therapeutics, telehealth for accessibility, and fostering interdisciplinary collaboration will establish “stress management” as a fundamental pillar of oncology care, shifting from tumor-centric treatment to holistic patient-centered strategies that improve survival and quality of life.

## Data Availability

The data that support the findings of this study are available from the corresponding authors upon reasonable request.
